# Phenotype-Specific Profiles of Isthmin-1, Trimethylamine N-Oxide, and Nitric Oxide in Polyendocrine Metabolic Ovarian Syndrome (Formerly PCOS): An Exploratory Biomarker Study

**DOI:** 10.3390/metabo16070488

**Published:** 2026-07-11

**Authors:** Alihan Tigli, Yakup Baykus, Rulin Deniz, Guzide Ece Akinci, Nazli Sener, Yasemin Ercan Degirmenci, Oguzhan Karakoc, Muhammet Bora Uzuner, Sefer Ustebay, Sermin Kilic, Engin Korkmazer, Murat Erdemir, Suleyman Aydin

**Affiliations:** 1 Department of Obstetrics and Gynecology, Faculty of Medicine, Bandırma OnYedi Eylül University, 10200 Balıkesir, Turkey; atigli@bandirma.edu.tr (A.T.); rdeniz@bandirma.edu.tr (R.D.); nsener@bandirma.edu.tr (N.S.); ydegirmenci@bandirma.edu.tr (Y.E.D.); 2 Gynecology and Obstetrics Clinic, Şehit Prof. Dr. Ilhan Varank Sancaktepe Training and Research Hospital, 34785 Istanbul, Turkey; geceakinci@gmail.com; 3 Gynecology and Obstetrics Clinic, Bandırma Training and Research Hospital, 10200 Balıkesir, Turkey; droguzhankarakoc@gmail.com; 4 Department of Anatomy, Faculty of Medicine, Bandırma OnYedi Eylül University, 10200 Balıkesir, Turkey; muzuner@bandirma.edu.tr; 5 Department of Pediatrics, Faculty of Medicine, Bandırma OnYedi Eylül University, 10200 Balıkesir, Turkey; sustebay@bandirma.edu.tr; 6 Gynecology and Obstetrics Clinic, Fethi Sekin City Hospital, 23280 Elazığ, Turkey; drserminyusufbilal23@gmail.com; 7 Private Clinic of Obstetrics and Gynecology, Barış Mah., Sumer St., No. 2, Sinanoglu Block, Floor 1, Flat No. 3, 16210 Bursa, Turkey; enginkorkmazer@gmail.com; 8 Department of Obstetrics and Gynecology and IVF, Bursa Jinemed IVF Centre, 16050 Bursa, Turkey; drmuraterdemir@gmail.com; 9 Department of Medical Biochemistry, Faculty of Medicine, Fırat University, 23119 Elazig, Turkey; saydin1@hotmail.com

**Keywords:** polyendocrine metabolic ovarian syndrome, polycystic ovary syndrome, phenotypes, trimethylamine N-oxide, nitric oxide, isthmin-1

## Abstract

**Background**: This study aimed to evaluate the phenotype-specific profiles of serum Isthmin-1 (ISM-1), Trimethylamine N-Oxide (TMAO) and Nitric Oxide (NO) levels in women diagnosed with Polyendocrine Metabolic Ovarian Syndrome (PMOS, formerly known as Polycystic Ovary Syndrome—PCOS) according to the Rotterdam criteria. **Methods:** This cross-sectional study enrolled 90 reproductive-aged women, divided equally into five groups (*n* = 18 per group) with similar baseline metabolic parameters: healthy controls and PMOS Phenotypes A, B, C, and D. To minimize confounding effects, individuals with recent use of specific medications were excluded, and 24 h dietary recalls were obtained. Fasting blood samples were collected during the early follicular phase. Serum ISM-1, TMAO, and NO levels were quantified via ELISA, and insulin resistance was determined using the HOMA-IR index. Data were adjusted for potential confounders, including age, BMI, and smoking status, using multivariate linear regression models. **Results**: No statistically significant differences were observed between the groups in key parameters such as BMI and HOMA-IR. Serum ISM-1 levels did not show a significant difference between the groups (*p* = 0.501). In contrast, NO levels were found to be significantly lower in all PMOS phenotypes compared to the control group (*p* < 0.001), and this reduction remained independent in regression models. TMAO levels, however, exhibited a phenotype-specific distribution; in the non-hyperandrogenic Phenotype D, they were found to be significantly lower than in the control group and hyperandrogenic phenotypes A and B. In the multivariate regression analysis, it was confirmed that Phenotype D was independently associated with low TMAO levels (B = −0.131, *p* = 0.027). **Conclusions:** Although PMOS patients share a similar profile of obesity and insulin resistance, they exhibit marked biochemical heterogeneity. Whilst the reduction in NO levels may indicate a generalised vascular change affecting all phenotypes, the observation of low TMAO levels specifically in the non-hyperandrogenic Phenotype D highlights a distinct biochemical signature associated with this subgroup, observed in the absence of hyperandrogenism. Our findings support the notion that adopting phenotype-specific, individualised approaches in the management of PMOS may be beneficial.

## 1. Introduction

Polyendocrine Metabolic Ovarian Syndrome (PMOS, formerly Polycystic Ovary Syndrome—PCOS) is a heterogeneous endocrine disorder characterized by clinical, hormonal, and metabolic components that affect women of reproductive age [1,2,3]. Following a recent international consensus statement, the terminology was updated to PMOS to more accurately reflect the complex metabolic facets of the syndrome [3]. Its community prevalence is reported to range between 5% and 15% [3]. Although classically characterized by ovulatory dysfunction, hyperandrogenism, and a polycystic ovary ultrasound image, it is no longer considered a purely reproductive pathology. Metabolic disorders such as insulin resistance, dyslipidaemia, low-grade chronic inflammation, oxidative stress and increased cardiometabolic risk are recognised as key components that contribute to the clinical burden of the syndrome [4]. Consequently, characterizing underlying metabolic and biochemical variations, rather than relying solely on diagnostic criteria, is essential for a comprehensive evaluation of PMOS.

According to the Rotterdam criteria, PMOS is categorized into four distinct phenotypes based on varying combinations of hyperandrogenism, ovulatory dysfunction, and a polycystic ovary ultrasound image [5,6]. Phenotype A represents the classic phenotype presenting with all three criteria; Phenotype B is characterized by hyperandrogenism and ovulatory dysfunction; Phenotype C denotes the ovulatory phenotype featuring hyperandrogenism and a polycystic ovary ultrasound image; and Phenotype D represents the non-hyperandrogenic phenotype, characterized by ovulatory dysfunction and a polycystic ovary ultrasound image in the absence of hyperandrogenism.

The clinical and metabolic risk profiles of these phenotypes diverge substantially. Evidence suggests that the hyperandrogenic phenotypes (A, B, and C) are associated with more pronounced metabolic impairment, insulin resistance, and cardiometabolic risk, whereas Phenotype D typically exhibits a more favorable metabolic risk profile [7,8,9]. This disparity implies that PMOS cannot be encapsulated by a single biochemical signature, highlighting the clinical relevance of phenotype-based stratifications.

Investigating novel biomarkers capable of elucidating the metabolic discrepancies among PMOS phenotypes may offer deeper insights into the heterogeneous nature of the syndrome. In this context, Trimethylamine N-Oxide (TMAO) has emerged as a metabolite of interest. TMAO is generated by the gut microbiota from dietary precursors—namely choline, phosphatidylcholine, and carnitine—and is subsequently oxidized in the liver by flavin-containing monooxygenase enzymes [10]. However, it is crucial to acknowledge that circulating TMAO concentrations are not solely dependent on microbiota composition; they are profoundly influenced by dietary intake, renal clearance, hepatic flavin-containing monooxygenase (FMO3) activity, and the overall metabolic status of the host. Although the literature frequently links elevated TMAO levels to atherosclerosis and cardiovascular risk [11,12], its potential involvement in the pathogenesis of insulin resistance and gut microbiota dysbiosis has increasingly garnered attention in recent years. Given the high prevalence of insulin resistance and inflammatory pathways in PMOS, it has been hypothesized that alterations in the gut microbiota may modulate circulating TMAO levels [13]. However, empirical data regarding how TMAO concentrations fluctuate in relation to the specific metabolic burdens across distinct PMOS phenotypes remain sparse.

Nitric oxide (NO) is a biologically unstable signaling molecule with a half-life measured in seconds; it exerts crucial regulatory effects on vascular tone, endothelial function, inflammation, and the oxidative/nitrosative balance [14]. The interplay among endothelial dysfunction, oxidative stress, and hyperandrogenism in PMOS has been a subject of long-standing investigation [15,16]. A recent meta-analysis demonstrated that NO bioavailability is significantly diminished in patients with PMOS, which may contribute to endothelial dysfunction [17]. Conversely, several studies have reported paradoxically elevated NO levels in patients with PMOS compared to healthy controls, suggesting that disruptions in the vasodilator-vasoconstrictor equilibrium may instead drive endothelial impairment [18]. These discordant findings in the literature are likely attributable to the heterogeneous nature of PMOS and the underlying pathophysiological discrepancies between hyperandrogenic and non-hyperandrogenic phenotypes. However, previous studies often analyzed PMOS as a single entity without phenotypic stratification. By systematically evaluating NO levels across distinct Rotterdam phenotypes with strictly matched baseline metabolic parameters, the present study aims to clarify whether these conflicting findings stem from the inherent phenotypic heterogeneity of PMOS and to determine if specific phenotypes exhibit distinct NO profiles.

Isthmin-1 (ISM-1) is a relatively novel adipokine-like molecule implicated in metabolic regulation, adipose tissue function, glucose homeostasis, and inflammatory pathways [19,20]. ISM-1 has been shown to enhance insulin sensitivity and stimulate glucose uptake, while also exerting regulatory effects on lipid metabolism and hepatic steatosis [21,22]. In PMOS, where metabolic dysfunction and insulin resistance are prominent features, evaluating ISM-1 concurrently with other metabolic and oxidative markers—such as TMAO and NO—may offer a holistic perspective on the biochemical heterogeneity of the syndrome.

Although numerous studies in the existing literature have focused on metabolic and inflammatory markers associated with PMOS, the specific distribution of ISM-1, TMAO, and NO levels across different PMOS phenotypes remains to be fully elucidated. Given the heterogeneous nature of PMOS, investigating inter-phenotypic biomarker variations could significantly contribute to a better understanding of the underlying pathophysiological mechanisms of the disease. The primary hypothesis of this study is that serum ISM-1, TMAO, and NO levels may differ significantly among PMOS phenotypes due to the distinct vascular and metabolic burden profiles inherent to hyperandrogenic and non-hyperandrogenic subgroups. Accordingly, this study aimed to compare ISM-1, TMAO, and NO levels between healthy women and those with four distinct PMOS phenotypes, and to evaluate the associations between these biomarkers and clinical, hormonal, and metabolic parameters.

## 2. Materials and Methods

### 2.1. Study Design and Sample

This observational, analytical, and comparative cross-sectional study was designed to evaluate serum ISM-1, NO, and TMAO levels across different PMOS phenotypes. The study was conducted among reproductive-aged women who attended the Obstetrics and Gynecology Outpatient Clinic at Bandırma Onyedi Eylül University Hospital between March 2025 and February 2026. A total of 90 women were enrolled and divided equally into five groups (n = 18 per group): a healthy control group and four distinct PMOS phenotype groups.

The study’s sample size was assessed using a post hoc power analysis performed with G*Power 3.1 software, taking into account the effect sizes of similar biomarker studies in the literature. Accordingly, to detect a moderate effect size (Cohen’s f = 0.40) among five groups at a significance level of α = 0.05, a total sample size of 90 participants provided a statistical power greater than 80%. The resulting study cohorts comprised a healthy control group (showing no evidence of PMOS) and Phenotypes A, B, C, and D.

A diagnosis of PMOS was established in accordance with the Rotterdam criteria [23], which require the presence of at least two of the following three features: oligo/anovulation, clinical and/or biochemical hyperandrogenism, and a polycystic ovary ultrasound image. Phenotype A comprises the group in which oligo/anovulation, hyperandrogenism, and a polycystic ovary ultrasound image are present together; Phenotype B comprises the group exhibiting oligo/anovulation and hyperandrogenism; Phenotype C comprises the group exhibiting hyperandrogenism and a polycystic ovary ultrasound image; and Phenotype D comprises the group exhibiting oligo/anovulation and a polycystic ovary ultrasound image, but in which hyperandrogenism was not detected. The control group consisted of healthy women with regular menstrual cycles, had no known metabolic or endocrine disorders, and met none of the PMOS diagnostic criteria. The slightly higher median age in the control group compared with the phenotype groups may reflect the selection of individuals with established reproductive maturity and clinically confirmed absence of PMOS. To eliminate potential age-related confounding, age was included and controlled as a covariate in all multivariate statistical analyses.

**Figure 1:**
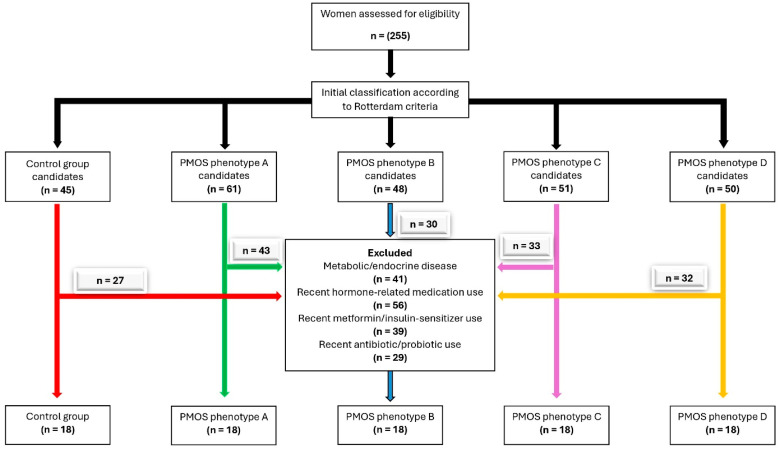
Participant flow diagram of the study population.

To enhance methodological reliability and eliminate potential confounding effects on the targeted biomarkers (particularly TMAO and NO), stringent exclusion criteria were enforced. Patients who had received oral contraceptives, insulin-sensitizing agents (e.g., metformin), anti-androgenic medications, or glucocorticoid therapy within the past 3 months were excluded. Additionally, individuals with a recent history of antibiotic or probiotic use, which could alter the gut microbiota and subsequently confound TMAO production, were excluded. Adhering to these inclusion and exclusion criteria, the enrollment and allocation process for the final sample (n = 90) is summarized in the participant flow diagram (Figure 1).

### 2.2. Data Collection Method

Data were recorded using a standard data collection form developed by the researchers. This form was used to assess participants’ demographic characteristics, clinical information, lifestyle variables, obstetric characteristics, PMOS phenotype and laboratory parameters. To ensure the standardisation of hormonal and biochemical assessments, blood samples were collected from all participants during the early follicular phase of the menstrual cycle (between days 2 and 5) and between 08:00 and 10:00 in the morning following a 10–12 h overnight fast.

Demographic and clinical variables included age, body mass index (BMI), smoking status, alcohol consumption, educational level and parity. Parity was categorised into two groups: nulliparous and parous. Educational level was classified as high school or below and university or above. Laboratory variables recorded included oestradiol (E2), follicle-stimulating hormone (FSH), luteinising hormone (LH), prolactin, total testosterone, fasting glucose, fasting insulin, high-density lipoprotein (HDL), low-density lipoprotein (LDL), triglycerides (TG), total cholesterol, HbA1c, and thyroid-stimulating hormone (TSH). In addition, although sex hormone-binding globulin (SHBG) is not included in the routine panel, total testosterone levels were used to assess hyperandrogenism, and the Homeostatic Model Assessment of Insulin Resistance (HOMA-IR) index was calculated to determine insulin resistance, a key metabolic component of PMOS [HOMA-IR = (Fasting insulin (µIU/mL) × Fasting glucose (mg/dL))/405] [24]. In addition, participants’ levels of physical activity were recorded and their 24 h dietary records were documented in detail—particularly regarding their intake of choline and red meat—in order to control for potential confounding factors that could directly affect the bioavailability of TMAO and NO.

### 2.3. Collection and Storage of Blood Samples

Venous blood samples collected during routine clinical evaluations were utilized for this study. Serum was separated following centrifugation. After the biochemical and hormonal parameters required for routine diagnostic assessment had been analyzed, the remaining serum samples were set aside for the determination of ISM-1, NO and TMAO levels. To prevent degradation of proteins and metabolites, these serum samples were divided into aliquots, transferred to Eppendorf tubes to avoid repetitive freeze–thaw cycles, and temporarily stored at −20 °C until the day of analysis. All assays were performed within the shortest possible timeframe post-collection to ensure sample stability.

### 2.4. Quantification of ISM-1, TMAO and NO

Serum ISM-1, TMAO, and NO levels were quantified via enzyme-linked immunosorbent assay (ELISA) in strict accordance with the manufacturers’ instructions. For all commercial kits utilized, the manufacturer-reported intra-assay and inter-assay coefficients of variation (CV) were below 10%. Serum ISM-1 levels were measured using the Human ISM-1 ELISA Kit (YLbiont, Shanghai, China, Catalogue No: YLA4565HU, 96 tests). The kit is based on the sandwich enzyme immunoassay principle and is designed for the quantitative measurement of ISM-1 levels in human serum, plasma, tissue homogenates and other biological fluids. Although liquid chromatography-tandem mass spectrometry (LC-MS/MS) is the gold standard method for TMAO measurement, the ELISA method was preferred in our study—as in many similar biomarker studies in the literature—due to its cost-effectiveness, accessibility and practicality in screening studies. Serum TMAO levels were measured using the Human Trimethylamine-N-oxide (TMAO) Kit (SunRed Biotechnology, Shanghai, China, Catalogue No: 201-12-7378, 96 tests). Because TMAO is a small molecule, the classic sandwich method is not suitable; therefore, this assay fundamentally operates on the principle of a competitive enzyme immunoassay. In this method, free TMAO in the sample competes with a fixed amount of labeled TMAO for binding sites on the specific antibodies pre-coated on the microplate. Furthermore, as native NO is a highly unstable gas with a half-life measured in seconds, its direct measurement is not feasible. Consequently, our study utilized the Human Nitric Oxide (NO) Assay Kit (SunRed Biotechnology, Catalogue No: 201-12-1511) to indirectly quantify total stable NO end-metabolites (nitrates and nitrites). The sample preparation and assay principle for this kit are based on the colorimetric Griess method, wherein nitrates in the biological fluid are first enzymatically reduced to nitrites via nitrate reductase, and the accumulated nitrites subsequently react with the Griess reagent (sulfanilamide and naphthylethylenediamine) to form a measurable colorimetric product at 540 nm.

All kits were used for research purposes only and were not used for diagnostic or therapeutic purposes. Measurements were carried out in accordance with the manufacturer’s protocols. Optical density readings of the microplates were taken using a microplate reader fitted with a 450 nm filter for the ISM-1 and TMAO assays, and a 540 nm filter for the NO assay, as recommended in the respective kit protocols.

### 2.5. Statistical Analysis

Statistical analysis of the data was performed using the SPSS software package (version 26.0). The distribution characteristics of continuous variables were assessed using the Shapiro–Wilk test. Continuous variables that did not follow a normal distribution were presented as the median and 25th–75th percentile values (IQR). Categorical variables were expressed as counts and percentages. The Kruskal–Wallis test was used to compare continuous variables between the control group and the PMOS phenotypes. For variables where a significant difference was detected in the Kruskal–Wallis analysis, pairwise comparisons were performed using the Mann–Whitney U test. To prevent an increase in Type I errors in the pairwise comparisons, a Bonferroni correction was applied; however, given the overly conservative nature of the Bonferroni correction, the statistical reliability of the findings was also verified using the Benjamini–Hochberg False Discovery Rate (FDR) method. In additional analyses comparing the control group with all PMOS cases together, the Mann–Whitney U test was used. The relationships between ISM-1, TMAO and NO levels and clinical, hormonal and metabolic parameters were assessed using Spearman’s correlation analysis. To control for false positives that might arise from multiple correlation tests, the significance thresholds were adjusted using the Benjamini–Hochberg FDR method.

To rigorously evaluate whether the observed variations in serum TMAO and NO levels across the study groups were independent associations rather than artifacts of potential demographic or lifestyle confounders, multivariate linear regression analyses were performed. Due to their distribution characteristics, TMAO and NO values were included in the model following logarithmic transformation. Model assumptions were tested prior to the regression analyses. The absence of multicollinearity among the independent variables was confirmed by calculating the Variance Inflation Factor (VIF < 5) and tolerance values (Tolerance > 0.2). The normality of the model residuals was verified using the Shapiro–Wilk test and Q-Q plots. In the regression analyses, logTMAO and logNO were taken as dependent variables. PMOS phenotypes were included in the model with the control group as the reference category. The models were adjusted for age, BMI, smoking status, alcohol consumption, parity and educational level. Regression results were presented as the unstandardised regression coefficient (B), standard error, standardised regression coefficient (β), model explanatory power (R^2^) and *p*-value. A *p*-value of <0.05 was considered statistically significant in all analyses.

## 3. Results

The baseline clinical, hormonal, and metabolic characteristics of the healthy control group and the PMOS phenotypes were compared. No statistically significant differences were observed among the groups regarding age, BMI, HOMA-IR, E2, prolactin, fasting glucose, fasting insulin, HDL, LDL, triglycerides, total cholesterol, HbA1c, or TSH levels. Although the median age of the healthy control group appeared higher than that of the PMOS phenotypes, this difference was not statistically significant across the five groups (*p* = 0.383). This is due to the non-parametric nature of the Kruskal–Wallis test, which evaluates mean ranks across overlapping distributions rather than absolute medians. Furthermore, to eliminate any potential age-related confounding, age was strictly controlled for as a covariate in all multivariate regression models. However, statistically significant differences were detected across the cohorts regarding LH and total testosterone levels (*p* = 0.004 and *p* < 0.001, respectively) (Table 1).

When comparing the healthy control group against the pooled PMOS cohort, the Mann–Whitney U test revealed no statistically significant differences in serum ISM-1 or TMAO levels between the two cohorts (*p* = 0.587 and *p* = 0.390, respectively). Conversely, serum NO levels were significantly lower in the pooled PMOS cohort compared with healthy controls (*p* < 0.001) (Table 2).

In a five-group analysis conducted to assess the metabolic heterogeneity of PMOS (Kruskal–Wallis test), no significant difference was observed between the control group and the PMOS phenotypes in terms of ISM-1 levels (*p* = 0.501). Whilst TMAO levels did not show a significant difference compared to the control group across the entire PMOS group (Table 2), a statistically significant difference was detected between the groups in the phenotype-based analysis (*p* < 0.001). This suggests that TMAO levels vary not so much due to the general presence of the syndrome, but rather due to metabolic heterogeneity between phenotypes. A significant difference between phenotypes was also observed in terms of NO levels (*p* < 0.001) (Table 3).

Following the detection of significant intergroup differences in serum TMAO and NO levels via the Kruskal–Wallis test, pairwise post hoc comparisons were performed using Bonferroni correction. The pairwise analyses revealed that serum NO levels were significantly higher in the healthy control group compared to both Phenotype A and Phenotype B (adjusted *p* < 0.001 and *p* < 0.001, respectively). Regarding TMAO profiles, Phenotype D exhibited significantly lower concentrations than the healthy control group, Phenotype A, and Phenotype B (adjusted *p* = 0.010, *p* < 0.001, and *p* < 0.001, respectively). Furthermore, serum NO levels were significantly higher in Phenotype D than in Phenotype A (adjusted *p* = 0.030). The raw, Bonferroni-adjusted, and FDR-adjusted *p*-values for all pairwise comparisons across the study groups are detailed in Table 4.

**Figure 2:**
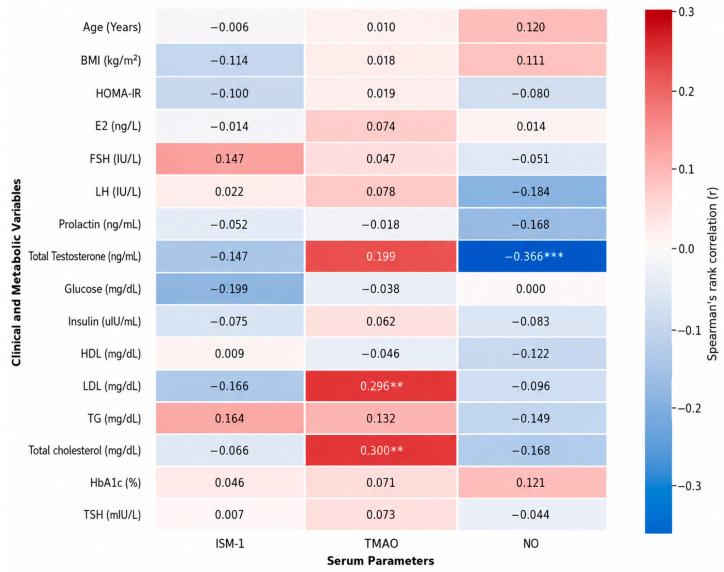
Heatmap illustrating Spearman’s rank correlations between serum ISM-1, TMAO, and NO levels and clinical, hormonal, and metabolic parameters. The data were analysed across the entire study population (n = 90). ** *p* < 0.01, *** *p* < 0.001 indicate statistically significant correlations. ISM-1: Isthmin-1; TMAO: trimethylamine N-oxide; NO: nitric oxide; BMI: body mass index; E2: oestradiol; FSH: follicle-stimulating hormone; LH: luteinising hormone; HDL: high-density lipoprotein; LDL: low-density lipoprotein; TG: triglycerides; HbA1c: glycated haemoglobin; TSH: thyroid-stimulating hormone; HOMA-IR: homeostatic model assessment of insulin resistance.

The relationships between serum ISM-1, TMAO, and NO levels and clinical, hormonal, and metabolic parameters were evaluated using Spearman’s rank correlation analysis. No statistically significant correlations were observed between ISM-1 levels and any of the investigated parameters. Conversely, serum TMAO levels demonstrated a significant positive correlation with both total cholesterol and LDL cholesterol (r = 0.300, *p* = 0.004 and r = 0.296, *p* = 0.005, respectively). Additionally, serum NO levels showed a significant negative correlation with total testosterone concentrations (r = −0.366, *p* < 0.001) (Figure 2).

Multivariate linear regression analyses were performed to identify independent predictors of serum TMAO and NO levels. The regression model for TMAO was statistically significant (F = 2.752, *p* = 0.006, R^2^ = 0.258 and adjusted R^2^ = 0.165). After adjusting for age, BMI, smoking status, alcohol consumption, parity, and educational attainment, serum TMAO levels were significantly lower in Phenotype D compared with the healthy control group (B = −0.131, *p* = 0.027). Among the covariates, smoking status demonstrated a significant negative association with TMAO levels (B = −0.111, *p* = 0.048).

The regression model for NO was also statistically significant (F = 5.194, *p* < 0.001, R^2^ = 0.397 and adjusted R^2^ = 0.320). Following multivariate adjustment, serum NO levels were significantly lower across all PMOS cohorts—Phenotypes A, B, C, and D—compared with the healthy control group. Notably, although the univariate post hoc pairwise comparison revealed no significant difference between the control group and Phenotype C, this multivariate regression model—which accounted for age, BMI, and other potential confounding factors—confirmed that Phenotype C was indeed independently associated with significantly reduced NO levels compared with healthy controls (B = −0.436, *p* = 0.001). Among the covariates, BMI exhibited a significant positive association with logNO (B = 0.016, *p* = 0.015) (Table 5).

## 4. Discussion

This study evaluated serum ISM-1, TMAO, and NO levels across distinct PMOS phenotypes—adopting the updated terminology—and investigated the relationships between these biomarkers and clinical, hormonal, and metabolic parameters. The primary findings of this investigation can be summarized into three main points: First, serum ISM-1 levels did not differ significantly between healthy controls and PMOS phenotypes. Second, serum NO levels were significantly and consistently lower across all PMOS phenotypes compared with healthy controls. Thirdly, and notably, TMAO levels exhibited a phenotype-specific distribution rather than being associated with the general presence of PMOS; in particular, they were found to be significantly lower in the non-hyperandrogenic Phenotype D compared with healthy controls and hyperandrogenic Phenotypes A and B.

### 4.1. NO and the Endothelial-Androgenic Axis in PMOS Phenotypes

One of the most prominent findings of this study is the marked reduction in serum NO levels within the PMOS cohort. The complex interplay among endothelial dysfunction, elevated oxidative stress, and chronic low-grade inflammation in PMOS is well established [25]. In the literature, it has been suggested that NO deficiency may be a significant mechanism in ovulatory dysfunction in PMOS, and that increased levels of reactive oxygen species (ROS) reduce NO bioavailability by inhibiting endothelial NOS [26]. In this context, the reduced NO levels observed across all PMOS phenotypes in our study indicate a significant biochemical abnormality reflecting the vascular and metabolic components of the syndrome. To fully appreciate the broad consequences of this reduction, the tissue specificity of NO synthases (eNOS, iNOS, and nNOS) and the complex network of NO-dependent enzymes must be considered. In humans, NO exerts its profound physiological and pathological effects by interacting with various downstream targets, including soluble guanylate cyclase (mediating vasodilation), cytochrome c oxidase (modulating mitochondrial respiration), caspases (regulating apoptosis), ribonucleotide reductase (affecting DNA synthesis), and both secreted and membrane-bound metalloproteinases (influencing tissue and follicular remodeling). Consequently, the substantial decrease in NO bioavailability observed in our PMOS cohorts may not only drive classical endothelial dysfunction but could also disrupt this extensive array of intracellular NO-dependent enzyme systems, thereby exacerbating the complex metabolic and reproductive burden of the syndrome.

The negative correlation observed between serum NO and total testosterone levels supports a potential interaction between the androgenic axis and vascular function. Hyperandrogenism can impair endothelial function and accelerate NO degradation via oxidative stress pathways [27]. However, the persistence of diminished NO levels in the non-hyperandrogenic Phenotype D in our study suggests that this pathophysiological process cannot be attributed solely to hyperandrogenism. While it could be hypothesized that adipose tissue dysfunction, insulin resistance, and subclinical inflammation underlie this reduction [28], such a mechanism remains speculative and cannot be directly supported by our data, given that BMI, fasting glucose, and fasting insulin levels were statistically balanced across all study cohorts.

Data regarding circulating NO levels in PMOS remain highly heterogeneous in the literature. While Kıran et al. reported elevated NO levels attributed to a vasodilator imbalance [18], a recent meta-analysis by Bahreiny et al. and an earlier report by Meng et al. demonstrated a distinct reduction in NO bioavailability in patients with PMOS [17,29]. These discrepant findings are likely due to variations in patient selection, sample characteristics (e.g., serum vs. plasma), the omission of phenotypic stratification, and methodological differences in quantification [29]. Furthermore, the healthy-control median serum NO concentration observed in the present study (257.70 µmol/L) is markedly higher than classical Griess-based reference values typically reported in healthy adults, which generally range between 20 and 60 µmol/L (e.g., Moshage et al.: 19.7 µmol/L; Giovannoni et al.: 32.8 µmol/L) [30,31]. This discrepancy between our absolute values and classical reference ranges likely stems from two principal factors: (i) the profound sensitivity of the colorimetric Griess assay to dietary nitrate intake, compounded by the absence of a controlled, multi-day low-nitrate diet prior to blood sampling in our study; and (ii) potential assay-specific calibration differences inherent to the commercial kit utilized. Nevertheless, despite these elevated absolute baselines, the directional finding of our study—a significant and consistent reduction in serum NO bioavailability across all PMOS phenotypes compared to controls—is biologically plausible and supported by previous meta-analytical data. Conversely, the positive association detected between BMI and logNO in our multivariate regression analysis was unexpected, considering the well-established link between obesity and endothelial impairment. This finding may stem from the relatively narrow BMI distribution in our cohort or a specific compensatory mechanism driven by variations in body composition.

### 4.2. TMAO, Phenotype-Specific Metabolic Burden and the DOGMA Theory

In contrast to the generalized reduction in NO, serum TMAO levels exhibited a phenotype-specific pattern rather than a uniform alteration across the entire PMOS spectrum. While pooled analyses comparing total PMOS cases against healthy controls yielded no significant differences, phenotype-stratified analyses revealed that circulating TMAO levels were uniquely and significantly lower in the non-hyperandrogenic Phenotype D.

This distinct divergence in TMAO concentrations between the hyperandrogenic (Phenotypes A and B) and non-hyperandrogenic (Phenotype D) cohorts aligns with the Dysbiosis of Gut Microbiota (DOGMA) hypothesis used to explain PMOS pathogenesis [11]. According to the DOGMA theory, hyperandrogenism causes dysbiosis by directly altering the gut microbiota, and this in turn triggers increased systemic inflammation and TMAO production [11,13]. Because gut microbiome composition was not assessed in our study, this hypothesis remains speculative. However, a possible explanation is that the absence of hyperandrogenic stress in Phenotype D might help preserve gut microbiota integrity, which could potentially be associated with lower levels of dysbiosis-mediated metabolites such as TMAO [32].

In clinical literature, Phenotype D is frequently associated with a more favorable metabolic risk profile compared with classic hyperandrogenic phenotypes. However, the suppressed TMAO levels observed in Phenotype D within our cohort should not be oversimplified as a direct indication of “reduced metabolic risk.” Notably, baseline metabolic parameters—including BMI, HOMA-IR, glucose, insulin, and lipid profiles—did not differ significantly among the phenotypes in our study. However, because direct measurements of gut microbiota composition, intermediate microbial metabolites, renal function, or FMO3 activity were not obtained, our findings must be interpreted strictly as associations. Consequently, rather than providing definitive evidence of altered microbial metabolism, the observed reduction in TMAO in Phenotype D simply highlights a potential phenotype-specific association that operates independently of hyperandrogenism.

On the other hand, the significant positive correlation observed between circulating TMAO levels and both total cholesterol and LDL cholesterol further strengthens the hypothesis that TMAO may be intimately linked to lipid metabolism and heightened cardiometabolic risk [33]. In addition to this expected finding, the negative correlation observed between smoking and TMAO in the multivariate regression analysis is another unexpected yet clinically significant finding of our study. Evidence in the literature suggests that cigarette smoke constituents may alter gut microbiota composition, potentially affecting bacterial taxa involved in the conversion of dietary precursors such as choline and carnitine into trimethylamine, as well as downstream hepatic oxidation pathways. In interpreting TMAO levels, which can be influenced by numerous variables such as diet, antibiotic use and the microbiota [34], it should not be forgotten that lifestyle factors such as smoking can also act as significant confounding factors.

### 4.3. ISM-1 as an Exploratory Indicator

In skeletal muscle and adipose tissue, initial reports indicated that ISM-1 stimulates GLUT4 translocation and facilitates glucose uptake through an insulin-independent pathway that activates, leading the mTORC2/PI3K/AKT cascade, while concurrently suppressing hepatic de novo lipogenesis [35]. However, the intracellular signaling mechanisms of ISM1 remain incompletely resolved in PMOS. A phosphoproteomic study demonstrated that Ism1 and insulin share only 53% overlap in downstream signaling, with Ism1 ablation in mice reducing phosphorylation of the key Akt effectors and mTORC1 targets in skeletal muscle—leading the authors to propose a “non-canonical mechanism” that is not necessarily dependent on direct AKT phosphorylation [22]. Consistent with this mechanistic uncertainty, a comprehensive review of ISM1 biology has catalogued multi-pathway signaling effects, reporting that ISM1 induces ERK phosphorylation but exerts no significant effect on PKA, PDK1, or GSK3β signaling [20]. Collectively, these findings suggest that the metabolic effects previously attributed to direct AKT stimulation may instead be distributed across multiple tissue-specific signaling nodes. This emerging uncertainty necessitates a cautious interpretation of ISM-1’s exact biological role. Although previous reports indicate that circulating ISM-1 concentrations are diminished in obesity and metabolic dysfunction-associated steatotic liver disease (MASLD)—displaying an inverse correlation with insulin resistance [36]—discrepant data suggest that this trend may fluctuate depending on disease severity and staging. Indeed, several recent studies have documented paradoxically elevated ISM-1 levels in advanced stages of metabolic derangement, such as type 2 diabetes mellitus and its associated macrovascular complications, potentially reflecting a compensatory physiological response [37]. Given the complex metabolic underpinnings of PMOS, investigating the role of ISM-1 in this syndrome is biologically plausible.

Nevertheless, in our study, serum ISM-1 levels demonstrated no statistically significant differences between healthy controls and the various PMOS phenotypes. Furthermore, no significant correlations were observed between ISM-1 concentrations and any of the evaluated clinical, hormonal, or metabolic parameters. This lack of differentiation likely stems from the homogeneous distribution of baseline metabolic parameters—such as BMI, HOMA-IR, glucose, insulin, and HbA1c—across all cohorts in our study population. Because ISM-1 is fundamentally associated with insulin resistance and adiposity, strictly matching the study groups on these variables may have inadvertently removed the biological variation necessary to detect meaningful inter-group differences in circulating ISM-1 levels. Given that alterations in ISM-1 typically parallel profound shifts in insulin resistance and adiposity, its failure to emerge as a discriminatory biomarker in this relatively young cohort without overt metabolic decompensation aligns with expectations derived from the literature.

However, the absence of statistical significance should not be interpreted as definitive proof that this adipokine plays no role in the pathogenesis of PMOS. The current sample size may have provided limited statistical power to detect subtle inter-cohort variations. Consequently, the absence of significant differences in ISM-1 may reflect a Type II error rather than a true lack of association. Nonetheless, reporting this negative finding is highly valuable for the scientific community. In the quest for novel metabolic biomarkers in complex syndromes like PMOS, the transparent reporting of molecules with limited discriminatory value is vital to mitigating publication bias [38]. Furthermore, the possibility that the ELISA kit used in this study may have detected circulating inactive degradation products of ISM-1 rather than its biologically active forms cannot be ruled out. In future studies, the evaluation of broader PMOS subgroups with pronounced insulin resistance and obesity, and the use of advanced analytical methods capable of measuring the molecule’s tissue-specific active fractions, may clarify the potential biological and clinical relevance of ISM-1 in PMOS.

### 4.4. Clinical and Research Implications

The findings of this study suggest that biomarker assessments in PMOS may be limited if conducted solely using a ‘disease present/absent’ approach. Our TMAO findings, in particular, demonstrate that key metabolic discrepancies can be easily overlooked if phenotype-based stratifications are omitted. While a pooled analysis comparing the entire PMOS cohort against healthy controls yielded no significant differences in TMAO levels, the distinct divergence of Phenotype D in the stratified analysis supports the necessity of phenotypic subclassification in the biochemical assessment of PMOS [39].

Conversely, our NO findings point toward an entirely different biological axis. The consistent reduction in serum NO levels across all PMOS phenotypes compared with healthy controls suggests that NO reflects a more generalized alteration in vascular tone or nitrosative balance inherent to the syndrome. Although the negative correlation between total testosterone and NO levels highlights a potential contribution of the androgenic axis to this pathway, the persistence of diminished NO levels in the non-hyperandrogenic Phenotype D indicates that this mechanism is not exclusively driven by hyperandrogenism. While the literature suggests that this persistent reduction may be linked to adipose tissue dysfunction, subclinical inflammation, and insulin resistance [28], this mechanistic link remains largely hypothetical within the constraints of our data, given that BMI and HOMA-IR values were statistically balanced across our cohorts.

Rather than proposing TMAO, NO, or ISM-1 as immediate diagnostic or prognostic tools, our study suggests that these molecules are potential instruments for mapping the biochemical heterogeneity across PMOS phenotypes. Future multicenter, prospective studies utilizing larger sample sizes and gold-standard analytical validation methods—such as LC-MS/MS—will help clarify the precise role of these biomarkers in the cardiometabolic risk stratification of PMOS phenotypes.

### 4.5. Strengths and Limitations of the Study

Our study contributes to the literature by detailing the biochemical heterogeneity of the syndrome through a separate examination of the four phenotypes of PMOS, and by simultaneously evaluating three biomarkers—ISM-1, TMAO and NO—which represent different biological pathways. Ensuring equal participant numbers across groups maintains the statistical balance of the analyses, whilst the successful control of potential confounding factors such as age, BMI and smoking status using multivariate regression models reinforces the methodological reliability of the study.

Nevertheless, several limitations inherent to our study design and analytical processes warrant consideration. First, the cross-sectional design precludes our ability to establish definitive causal relationships between the evaluated biomarker profiles and PMOS phenotypes. Second, because this investigation was conducted at a single center, the generalizability of our findings to broader or diverse populations remains limited. Third, our reliance on a post hoc power calculation rather than a prospective sample size estimation is a notable methodological limitation. With only 18 participants per group, the study is likely underpowered to detect small to moderate differences in emerging biomarkers such as ISM-1, meaning the lack of significant findings may reflect a Type II error rather than a true absence of association. Therefore, negative findings must be interpreted with extreme caution. Additionally, although the higher median age in the healthy control group relative to the PMOS cohorts was statistically controlled for in our regression models, we cannot completely rule out unmeasured, age-dependent confounding effects on vascular and microbial biomarkers. Fourth, the phenotypic and anthropometric characterization of our cohort was incomplete. Specifically, reliance on total testosterone without assessing sex hormone-binding globulin (SHBG) and the free androgen index (FAI) may have limited the precise characterization of the hyperandrogenic status. Additionally, anti-Müllerian hormone (AMH), which has increasing relevance in PMOS phenotyping, was not measured. Furthermore, the absence of waist circumference measurements is a notable limitation, as BMI alone does not adequately capture visceral adiposity, which is intimately linked to metabolic dysfunction in PMOS. Fifth, while we utilized a 24 h dietary recall to monitor the intake of specific precursors, this single assessment may be insufficient to accurately capture habitual dietary exposure. Because circulating TMAO and NO (nitrate/nitrite) concentrations are highly sensitive to long-term dietary habits—such as the consumption of leafy vegetables, processed meats, and choline-rich foods—the lack of comprehensive dietary standardization and prolonged nutrient analysis remains a notable limitation that could introduce residual confounding. Sixth, the correlation analysis was performed on the pooled study population (n = 90). While this approach maximizes statistical power, it may inadvertently reflect inter-phenotypic variances rather than true within-group biological relationships. Although partially stratifying the data by combining the hyperandrogenic phenotypes (A + B + C; n = 54) against Phenotype D (n = 18) and controls (n = 18) was considered as an alternative approach, computing Spearman correlations across such highly unbalanced subsets—especially those with fewer than 20 subjects—carries a high risk of generating statistically unstable and misleading correlation coefficients. Because stratifying the analysis in this manner would severely compromise methodological integrity, we maintained the pooled approach; however, these pooled associations must be interpreted with caution.

Furthermore, specific analytical limitations regarding our quantification methods must be highlighted. For TMAO quantification, we utilized a commercial ELISA kit rather than liquid chromatography-tandem mass spectrometry (LC-MS/MS), which is the recognized gold standard. We acknowledge that the analytical validity of ELISA for small molecules like TMAO (~75 Da) is highly controversial due to potential cross-reactivity and specificity issues. Indeed, the absolute TMAO concentrations observed in our cohort (approximately 1100–1600 ng/mL) were substantially higher than fasting plasma levels typically reported in healthy populations using LC-MS/MS. This discrepancy likely reflects methodological cross-reactivity rather than true physiological elevations. Consequently, while the relative inter-group differences remain statistically significant within the context of the assay used, our absolute TMAO values must be interpreted with extreme caution. These results should be viewed strictly as hypothesis-generating and require future validation through LC-MS/MS before drawing definitive clinical conclusions. Similarly, because native NO is an extremely short-lived and unstable molecule, our reliance on evaluating its stable surrogate metabolites and storing samples under standard −20 °C conditions represent other potential analytical factors that could affect downstream concentrations.

Finally, the absolute magnitude of serum NO in the present healthy controls is higher than classical Griess reference values. As discussed, this discrepancy likely reflects the absence of a controlled dietary pre-phase and proprietary kit calibration. Therefore, while the relative PMOS–control differences observed in this study are internally consistent, the absolute concentrations should be interpreted with caution. Future studies should employ a second analytical platform (e.g., LC-MS/MS or chemiluminescence) to triangulate the actual magnitude of circulating NO in PMOS populations, and greater confidence should currently be placed in group-level comparative patterns.

## 5. Conclusions

This study suggests that it may be difficult to explain the complex metabolic and vascular underpinnings of PMOS using a single biochemical profile, and highlights the importance of considering phenotype-based approaches in the management of cardiometabolic risk associated with the syndrome. ISM-1 levels did not demonstrate a distinct discriminatory feature between phenotypes in this cohort. In contrast, whilst low levels of NO metabolites across all PMOS phenotypes indicate a general vascular alteration, the finding that TMAO levels were low only in the non-hyperandrogenic Phenotype D highlights a distinct biochemical profile observed in the absence of hyperandrogenism. These exploratory data support the concept of individualising disease management according to sub-phenotypes, and the potential role of the identified biomarker associations should be further investigated using direct microbiome assessments and advanced analytical techniques.

## Figures and Tables

**Table 1:** Basic clinical, hormonal and metabolic characteristics of the control group and PMOS phenotypes.

Variable	Control Group n = 18	PMOS Phenotype A n = 18	PMOS Phenotype B n = 18	PMOS Phenotype C n = 18	PMOS Phenotype D n = 18	*p*
Age (Years)	30.50 (22.50–34.25)	21.00 (19.00–24.50)	19.50 (18.75–22.00)	21.50 (18.75–23.75)	20.00 (18.75–27.50)	0.383
BMI (kg/m^2^)	22.60 (20.49–25.45)	24.57 (20.89–32.04)	26.30 (20.55–33.95)	26.76 (23.29–29.39)	23.47 (21.80–29.94)	0.399
HOMA-IR	1.43 (0.98–1.83)	2.41 (1.13–4.54)	2.66 (1.61–3.92)	2.49 (1.21–3.73)	1.75 (1.30–2.66)	0.133
E2 (ng/L)	36.00 (24.00–42.25)	44.50 (30.50–58.00)	35.00 (24.75–44.00)	39.00 (28.25–49.25)	29.50 (24.25–69.50)	0.461
FSH (IU/L)	5.90 (5.03–7.67)	5.70 (4.71–6.48)	5.18 (4.63–6.33)	4.99 (3.67–5.92)	4.92 (4.13–5.54)	0.096
LH (IU/L)	4.30 (3.19–6.15)	8.01 (5.52–12.30)	5.15 (4.10–6.47)	5.53 (3.33–8.09)	4.21 (3.66–5.95)	**0.004**
Prolactin (ng/mL)	23.77 (12.60–40.23)	17.83 (13.77–30.44)	20.52 (15.90–37.43)	20.72 (14.24–29.30)	18.23 (13.14–24.22)	0.539
Total Testosterone (ng/mL)	27.07 (23.36–35.98)	67.08 (62.52–74.08)	56.32 (46.36–72.12)	57.29 (54.51–73.94)	35.22 (25.57–36.59)	**<0.001**
Glucose (mg/dL)	90.00 (85.75–94.00)	89.00 (83.75–104.00)	94.00 (86.75–106.50)	94.50 (81.75–99.75)	90.50 (85.50–96.75)	0.561
Insulin (μIU/mL)	6.20 (4.58–8.23)	12.20 (5.75–17.28)	12.40 (7.18–15.35)	9.65 (5.85–16.98)	8.60 (5.90–13.00)	0.127
HDL (mg/dL)	54.00 (44.75–60.50)	55.00 (41.50–63.75)	53.50 (46.75–65.25)	58.50 (44.50–64.00)	54.50 (48.25–63.25)	0.957
LDL (mg/dL)	100.50 (82.75–122.50)	114.00 (87.75–129.00)	107.00 (74.25–115.25)	90.50 (82.00–113.25)	91.50 (77.75–105.25)	0.305
TG (mg/dL)	72.00 (60.50–87.50)	88.50 (65.50–181.00)	88.50 (58.50–128.00)	83.50 (63.25–138.25)	84.50 (71.00–127.25)	0.440
Total cholesterol (mg/dL)	164.00 (155.50–192.50)	193.00 (161.25–204.75)	191.00 (145.75–197.25)	166.50 (157.00–187.50)	167.50 (146.50–191.25)	0.336
HbA1c (%)	5.03 (4.84–5.20)	5.01 (4.79–5.22)	4.98 (4.85–5.30)	4.92 (4.70–5.15)	4.92 (4.64–5.09)	0.512
TSH (mIU/L)	2.07 (1.54–2.80)	2.31 (1.64–3.11)	1.93 (1.17–2.90)	1.83 (1.17–2.42)	1.35 (1.12–1.94)	0.147

Data are presented as the median (25th–75th percentile). The Kruskal–Wallis test was used for comparisons between groups. *p*-values in bold are statistically significant. BMI: body mass index; E2: oestradiol; FSH: follicle-stimulating hormone; LH: luteinising hormone; HDL: high-density lipoprotein; LDL: low-density lipoprotein; TG: triglycerides; HbA1c: glycated haemoglobin; TSH: thyroid-stimulating hormone; PMOS: polyendocrine metabolic ovarian syndrome. HOMA-IR: homeostatic model assessment of insulin resistance.

**Table 2:** Comparison of ISTHMIN, TMAO and NO levels between the control group and all PMOS cases.

Variable	Control n = 18	All PMOS n = 72	U	*p*
ISM-1 (ng/mL)	10.16 (9.19–12.22)	10.16 (9.19–11.13)	594.50	0.587
TMAO (ng/mL)	1493.33 (1258.67–1856.00)	1365.33 (1130.67–1856.00)	563.00	0.390
NO (µmol/L)	257.70 (174.50–398.55)	73.00 (34.18–150.03)	212.00	**<0.001**

Data are presented as the median (25th–75th percentile). The Mann–Whitney U test was used to compare the control group with all PMOS cases. A *p*-value in bold is statistically significant. U denotes the Mann–Whitney U test statistic. ISM-1: Isthmin-1; TMAO: trimethylamine N-oxide; NO: nitric oxide; PMOS: Polyendocrine Metabolic Ovarian Syndrome.

**Table 3:** Comparison of ISTHMIN, TMAO, and NO levels among study groups.

Variable	Control n = 18	Phenotype A n = 18	Phenotype B n = 18	Phenotype C n = 18	Phenotype D n = 18	*p*-Value
ISM-1 (ng/mL)	10.16 (9.19–12.22)	9.68 (9.19–10.65)	9.92 (9.19–11.25)	10.89 (9.92–12.70)	10.16 (9.19–11.73)	0.501
TMAO (ng/mL)	1493.33 (1258.67–1856.00)	1536.00 (1344.00–2602.67)	1578.67 (1450.67–2304.00)	1280.00 (917.33–1728.00)	1152.00 (938.67–1280.00)	<0.001
NO (µmol/L)	257.70 (174.50–398.55)	37.65 (31.83–89.93)	44.65 (31.83–154.25)	71.00 (38.65–280.85)	122.85 (69.50–254.43)	<0.001

Data are presented as median (25th–75th percentile). Comparisons among groups were performed using the Kruskal–Wallis test. ISM-1: isthmin-1; TMAO: trimethylamine N-oxide; NO: nitric oxide.

**Table 4:** Post hoc pairwise comparisons for TMAO and NO levels.

Variable	Pairwise Comparison	Direction of Difference	U	Raw *p*	Bonferroni adj. *p*	FDR adj. q
TMAO	Control vs. Pheno. A	Pheno. A > Control	136.000	0.409	1.000	0.454
TMAO	Control vs. Pheno. B	Pheno. B > Control	126.000	0.252	1.000	0.315
TMAO	Control vs. Pheno. C	Control > Pheno. C	115.500	0.140	1.000	0.233
TMAO	Control vs. Pheno. D	Control > Pheno. D	61.500	**0.001**	**0.010**	**0.003**
TMAO	Pheno. A vs. Pheno. B	Pheno. B > Pheno. A	159.500	0.937	1.000	0.937
TMAO	Pheno. A vs. Pheno. C	Pheno. A > Pheno. C	92.000	0.026	0.260	0.052
TMAO	Pheno. A vs. Pheno. D	Pheno. A > Pheno. D	41.000	**<0.001**	**<0.010**	**0.003**
TMAO	Pheno. B vs. Pheno. C	Pheno. B > Pheno. C	83.000	0.012	0.120	**0.030**
TMAO	Pheno. B vs. Pheno. D	Pheno. B > Pheno. D	28.500	**<0.001**	**<0.010**	**0.003**
TMAO	Pheno. C vs. Pheno. D	Pheno. C > Pheno. D	124.500	0.232	1.000	0.315
NO	Control vs. Pheno. A	Control > Pheno. A	14.000	**<0.001**	**<0.010**	**0.005**
NO	Control vs. Pheno. B	Control > Pheno. B	40.500	**<0.001**	**<0.010**	**0.005**
NO	Control vs. Pheno. C	Control > Pheno. C	78.000	0.008	0.080	**0.018**
NO	Control vs. Pheno. D	Control > Pheno. D	79.500	0.009	0.090	**0.018**
NO	Pheno. A vs. Pheno. B	Pheno. B > Pheno. A	128.000	0.282	1.000	0.321
NO	Pheno. A vs. Pheno. C	Pheno. C > Pheno. A	102.000	0.058	0.580	0.094
NO	Pheno. A vs. Pheno. D	Pheno. D > Pheno. A	67.500	**0.003**	**0.030**	**0.010**
NO	Pheno. B vs. Pheno. C	Pheno. C > Pheno. B	128.500	0.289	1.000	0.321
NO	Pheno. B vs. Pheno. D	Pheno. D > Pheno. B	104.000	0.066	0.660	0.094
NO	Pheno. C vs. Pheno. D	Pheno. D > Pheno. C	135.500	0.402	1.000	0.402

Pairwise comparisons were performed using the Mann–Whitney U test. Adjusted *p*-values were calculated using Bonferroni correction for 10 pairwise comparisons per biomarker. U: Mann–Whitney U statistic; Raw *p*: unadjusted *p*-value; Adj. *p*: adjusted *p*-value; FDR adj. q: adjusted false discovery rate q-value; Pheno.: phenotype; TMAO: trimethylamine N-oxide; NO: nitric oxide. Raw *p*, Bonferroni-adjusted *p*, and FDR-adjusted q values < 0.05 were considered statistically significant. A *p*-value in bold is statistically significant.

**Table 5:** Multivariate linear regression analysis for TMAO and NO levels.

Dependent Variable	Variable	B	SE	β	*p* Value
logTMAO	Pheno. A vs. Control	0.103	0.057	0.230	0.074
	Pheno. B vs. Control	0.072	0.057	0.161	0.211
	Pheno. C vs. Control	−0.044	0.058	−0.098	0.451
	Pheno. D vs. Control	−0.131	0.058	−0.293	**0.027**
	Age	0.005	0.005	0.117	0.277
	BMI	0.001	0.003	0.034	0.743
	Smoking	−0.111	0.055	−0.210	**0.048**
	Alcohol	−0.006	0.042	−0.016	0.884
	Parity	0.024	0.048	0.052	0.627
	Level of education	0.010	0.040	0.025	0.812
logNO	Pheno. A vs. Control	−0.743	0.122	−0.698	**<0.001**
	Pheno. B vs. Control	−0.621	0.123	−0.584	**<0.001**
	Pheno. C vs. Control	−0.436	0.124	−0.410	**0.001**
	Pheno. D vs. Control	−0.324	0.124	−0.304	**0.011**
	Age	0.005	0.010	0.048	0.620
	BMI	0.016	0.006	0.229	**0.015**
	Smoking	−0.170	0.118	−0.136	0.154
	Alcohol	0.001	0.091	0.001	0.990
	Parity	−0.096	0.103	−0.088	0.356
	Level of education	−0.028	0.086	−0.031	0.742

In the multivariate linear regression analysis, log-transformed TMAO and NO values were used as the dependent variables. The control group was taken as the reference category. For the logTMAO model, F = 2.752, *p* = 0.006, R^2^ = 0.258 and adjusted R^2^ = 0.165; for the logNO model, F = 5.194, *p* < 0.001, R^2^ = 0.397 and adjusted R^2^ = 0.320 were calculated. Bolded *p*-values are statistically significant. B: unstandardised regression coefficient; β: standardised regression coefficient; SE: standard error; BMI: body mass index; Pheno.: phenotype; TMAO: trimethylamine N-oxide; NO: nitric oxide.

## Data Availability

The data that support the findings of this study are available from the corresponding author upon reasonable request.
